# Mass Spectrometry: A Rosetta Stone to Learn How Fungi Interact and Talk

**DOI:** 10.3390/life10060089

**Published:** 2020-06-20

**Authors:** Erika Calla-Quispe, Hammerly Lino Fuentes-Rivera, Pablo Ramírez, Carlos Martel, Alfredo J. Ibañez

**Affiliations:** 1 Instituto de Ciencias Ómicas y Biotecnología Aplicada (ICOBA), Pontificia Universidad Católica del Perú (PUCP), Av. Universitaria 1801, San Miguel 15088, Lima, Peru; erika.callaq@pucp.edu.pe (E.C.-Q.); hammerly.lino@unmsm.edu.pe (H.L.F.-R.); carlos.martel@pucp.pe (C.M.); 2 Laboratory of Molecular Microbiology and Biotechnology, Faculty of Biological Sciences, Universidad Nacional Mayor de San Marcos (UNMSM), Av. Germán Amézaga 375, Lima 15081, Peru; pramirezr@unmsm.edu.pe; 3 Museo de Historia Natural, Universidad Nacional Mayor de San Marcos (UNMSM), Av. Arenales 1256, Jesús María 15072, Lima, Peru

**Keywords:** fungal metabolites, volatile organic compounds, non-volatile compounds, ecological interactions, biotyping, metabolomics

## Abstract

Fungi are a highly diverse group of heterotrophic organisms that play an important role in diverse ecological interactions, many of which are chemically mediated. Fungi have a very versatile metabolism, which allows them to synthesize a large number of still little-known chemical compounds, such as soluble compounds that are secreted into the medium and volatile compounds that are chemical mediators over short and long distances. Mass spectrometry (MS) is currently playing a dominant role in mycological studies, mainly due to its inherent sensitivity and rapid identification capabilities of different metabolites. Furthermore, MS has also been used as a reliable and accurate tool for fungi identification (i.e., biotyping). Here, we introduce the readers about fungal specialized metabolites, their role in ecological interactions and provide an overview on the MS-based techniques used in fungal studies. We particularly present the importance of sampling techniques, strategies to reduce false-positive identification and new MS-based analytical strategies that can be used in mycological studies, further expanding the use of MS in broader applications. Therefore, we foresee a bright future for mass spectrometry-based research in the field of mycology.

## 1. Introduction

Fungi comprise a monophyletic yet unresolved kingdom. At the moment, it is still not possible to diagnose fungi based on specific characters and they should be regarded as a group without synapomorphies [1]. Nevertheless, it is clear that the evolution of both osmotrophic and rigid cells, which are widespread among fungi, are among the evolutionary developments that allow their diversification and ecological success [1]. It is estimated that 1.5 to 3.8 million fungal species occur on Earth, despite only around 100,000 species being known to science [2,3,4,5]. This indicates that fungi are far more diverse than other kingdoms, and a perspective research area. Fungi have been very successful in inhabiting the world, and they can be found in terrestrial and aquatic environments, as symbiotic associations as well as saprophytic or parasitic organisms. Fungal ecological success is also a consequence of their huge metabolic machinery that allows them to produce diverse chemical compounds, which are important for their ecological interactions and survival [6,7,8,9].

**Figure 1:**
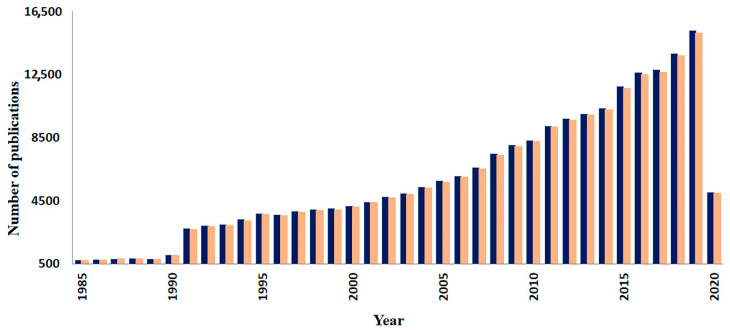
Development of fungal studies involving fungal metabolites and mass spectrometry. Publications from 1985 to 2020 involving metabolites (blue bars) and mass spectrometry (orange bars) in mycological studies. The search was carried out in the portal of the Web of Science using the following search terms: (i) “fungal OR fungi AND metabolite*”; and (ii) “fungal OR fungi AND mass spectromet*”.

Fungi produce a large diversity of secondary metabolites (hereafter referred as specialized metabolites), from which more than 15,600 are unique to them [10]. The production of specialized metabolites by fungi is an evolutionary advantage, as they use metabolites to defend themselves, takedown defenses, as well as to takedown defenses of their potential food sources, and for intra-/interspecific chemical communication [9,11,12,13]. For example, fungi communicate and interact within and with other species either via volatile organic compounds (VOCs) or non-volatile secreted compounds [14,15]. Most of these compounds have been identified using mass spectrometry (hereafter MS). Therefore, MS is a powerful tool in understanding fungi metabolism. Fungal metabolites are being continuously discovered and the rate of these discoveries has been accelerated significantly during the last two decades. This is thanks to the development of MS, which has been used in mycological studies such as biotyping, ecology and monitoring the production of predominantly specialized metabolites. Indeed, in a search within the Web of Science portal, we found that the number and pattern of scientific publications per year involving fungal metabolites are almost identical to the number and pattern of mycological studies involving MS techniques (Figure 1). The development of MS instruments and techniques during the last three decades has also stimulated the increasing number of publications related to fungal metabolites, which heavily increased from around 750 released publications in 1985 up to around 16,000 in 2019 (Figure 1).

The inherent sensitivity and rapid data acquisition capabilities of MS instruments, in addition to new MS-based analytical techniques, have thus prompted the further development of metabolomic studies on fungi. However, the use of cutting-edge MS instruments and techniques alone is not enough, if there are not clear analytical protocols that allow the researches to correctly address and achieve their study goals. Thereby, we believe that it is necessary and of current interest for a combination of sampling and analytical techniques used in MS-based fungal metabolomic studies. Our goal in this review is to show the versatility and potential use of MS-based techniques in mycological studies by providing information on the diverse metabolites known from fungi, their ecological role and the MS-based techniques used.

## 2. Fungi and Their Specialized Metabolites

### 2.1. Taxonomical Fungal Groups and Their Specialized Metabolites

#### 2.1.1. Lower Fungi

Lower fungi are an unnatural group that comprises diverse phyla of basal fungi [16]. Two unnatural groups are found here: zoosporic and zygomycetous fungi. Zoosporic fungi are the most basal ones and are characterized by producing zoospores that bear a single flagellum, so they are able to move, whereas zygomycetous fungi are characterized by developing a zygospore by fusion of specialized hyphae during sexual reproduction and the absence of septate hyphae [17]. Zoosporic fungi embrace diverse phyla such as Chytridiomycota, Blastocladiomycota, Neocallimastigomycota and the Opisthosporidia group, whereas zygomycetous fungi embrace the phyla Glomeromycota, Mucoromycota and Zoopagomycota [16,18]. Lower fungi are highly diverse in regard to their nutrition and lifestyle from heterotrophs and saprobes to symbiotes and parasites, and they can be found in terrestrial and aquatic environments [16,19,20]. Members of lower fungi are not well represented in mycological studies compared with those of higher fungi, and they have been believed to be incapable of producing specialized metabolites [21] because some previously reported compounds have been found out to be actually produced by endosymbiotic bacteria (e.g., rhizoxins and rhizonins; [22,23]) and some screened lower fungi did not have enzyme-encoding genes, which are key in specialized metabolites production (e.g., polyketide synthases, nonribosomal peptides synthetases, tryptophan synthases, and dimethylallyl tryptophan synthase; [24]). However, recent studies involving clusters of orthologous groups of proteins would indicate that zoosporic and zygomycetous fungi produce specialized metabolites, but their capacity is underdeveloped compared with higher fungi [10]. In fact, sterol compounds [25,26] have been recorded for them and zygomycetous fungi are known for their diversity of carotenoids [21,27]. Moreover, the identification of VOCs in some *Mucor* species (phylum Mucoromycota) also confirmed that they release volatile compounds [28,29,30]. Therefore, it is likely that many other lower fungi produce some specialized metabolites, and in particular VOCs, but they have not been examined or the appropriate sampling technique has not been used (e.g., extracts are not optimal for volatile collection, but instead headspace sampling; see below).

#### 2.1.2. Higher Fungi

Higher fungi or Dikarya contain the largest number of fungi described to date [16,31]. They were historically divided into two phyla (i.e., Ascomycota and Basidiomycota), although a third phylum (i.e., the Enthorrizomycota) was recently introduced [32]. Dikarya are mainly characterized by the absence of flagella on their gametes and the fact that their cells bear two genetically distinct haploid nuclei [33]. Members of Dikarya have evolved and developed very diverse lifestyles: some fungi evolved symbiotic associations with cyanobacteria (i.e., lichens) and with insects [34,35], others closely interact with plants, insects and bacteria [36,37,38]. Some species of this group biosynthesize compounds with biological activity against other fungi, bacteria and harmful insects and nematodes [39]. Due to well-developed machinery to synthesize specialized metabolites, higher fungi produce and store a variety of chemical compounds in them, e.g., mycelia and fruit bodies, to be used in diverse ecological interactions [40]. In addition, it is necessary to add that most of the specialized metabolites known from fungal origin belong to Ascomycota and Basidiomycota [9,10,11,41].

### 2.2. Classes and Ecological Role of Fungal Specialized Metabolites

#### 2.2.1. Classes of the Specialized Metabolites of Fungi

Fungi produce an array of specialized metabolites. To this date, more than 15,600 specialized metabolites are reported [10]; however, although new specialized metabolites are being constantly described, their number seems to be still far from the real fungal chemical diversity. Less attention has been directed to fungal VOCs and, only during the last two decades, the number of studies on fungal VOCs has been greatly increased [42,43,44] and over 500 newly discovered volatile compounds from 340 fungi species have been described [45]. The increasing knowledge related to fungal specialized metabolites is driven by their importance for industrial and non-industrial activities such as pharmacy and agriculture, among others (see [46]). Fungal specialized metabolites mainly contain C, H, O and N atoms, but may also include S, P, Cl, Br and F [11]; their chemical structure commonly contains polar functional groups such as hydroxyl, carboxyl, carbonyl and amino, among others. [11,41]. A variety of enzymes are involved in the synthesis of specialized metabolites in fungi, among others, such as nonribosomal peptide synthetases, polyketide synthases and terpene synthases [10,12,47,48]. Here, we provide information on the main fungal specialized metabolites such as alkaloids, nonribosomal peptides, polyketides, shikimic acid derived compounds and terpenoids.

##### Nonribosomal Peptides

These specialized metabolites are synthesized by the group of enzymes known as nonribosomal peptide synthetases. Nonribosomal peptides are structurally diverse as they are formed by proteinogenic and non-proteinogenic amino acids, which can be connected in a linear or cyclic fashion, and be further modified by tailoring enzymes [49,50,51]. Nevertheless, they have mostly macrocyclic structures, which can be carried out by cyclization of the N-terminal amino group and the carboxy terminus of the peptide chain [49]. Their diverse chemical features address the broad spectrum of biological activities in which they are involved [49,51]. Functions of nonribosomal peptides reach from immunosuppressive and cytostatic to antibiotic and toxic [49], and therefore some are used as pharmaceutical products such as the well-known penicillin, cephalosporin and cyclosporin.

##### Polyketides

Polyketides are the largest and most diverse group of fungal specialized metabolites [47]. They are derived of short-chain carboxylic acids, such as acetate and malonate, and are biosynthesized by large iterative multifunctional polyketide synthases [10,52,53]. The diversity found in polyketide structures is achieved by the action of these enzymes that can modify chain lengths, reduce methylate in different position and modify the polyketide backbone, in addition to tailoring enzymes that can further rearrange and modify poliketide structures [53]. Thus, polyketides can be as simple as monocyclic to complex polycyclic aromatic compounds [52]. Polyketides play an important role in promoting survival advantages of the host organisms and often have antibiotic and biocidal properties [52,53]. Especially toxic are the powerful mycotoxins such as citrinin, ochratoxins and the well-known aflatoxin, which are produced by *Aspergillus* species and can lead to immunotoxicity in humans.

##### Shikimic Acid Derived Compounds

These specialized metabolites, as its name suggest, are derived from the shikimic acid pathway. This pathway started with the condensation of phosphoenolpyruvate and erythrose 4-phosphate to give shikimic acid, and its final product is chorismate, which is the precursor of the synthesis for the amino acids: tryptophan, L-phenylalanine and L-tyrosine [10,54]. Chorismate is also the precursor of diverse fungal specialized metabolites such as pulvinones, terphenyl quinones, macrolides and strobilurins [8]. Although the shikimic acid pathway occurs in bacteria, plant and fungi, the distinct enzymatic processes in fungi make it different from that in plants and bacteria, and therefore structures of many shikimic acid-derived metabolites are particular to fungi [10]. Shikimic acid-derived compounds can function as pigments and biocides [8].

##### Terpenoids

Although plants are well-known because of their terpenoid diversity, higher fungi (Ascomycota and Basidiomycota) also produce a large variety of terpenoids. Cyclic terpenoids in fungi are synthetized by enzymes of the terpene cyclase family [10,30,47]. In fungi, terpenoids can be linear and cyclic, saturated and unsaturated compounds, with skeletons of two (i.e., monoterpenes), three (i.e., sesquiterpenes), four (i.e., diterpenes) and several isoprenes (e.g., carotenoids) [10,30,41,47]. In addition, specialized metabolites from other classes (e.g., polyketides, shikimic acid-derived compounds) are sometimes incorporated to terpenoids, resulting in compounds with a mixed biosynthetic origin [10,11]. Sesquiterpenoids and diterpenoids are dominant terpenoids among fungi [10]. Terpenoids can act as attractants, repellents, toxins and pigments and they are widely used in industry as flavors, fragrances, pharmaceuticals, food additives and cosmetics.

##### Alkaloids

These are specialized metabolites that bear at least one N atom in a heterocyclic ring structure [11,55]. Indole alkaloids are particularly abundant in fungi, especially among Ascomycota. Most indole alkaloids are derived from L-tryptophan, which is the indole donor [56]. Among fungal indole alkaloids, ergot alkaloids synthetized by *Claviceps purpurea* are the best understood as its biosynthesis pathway is known [47,55]. Indole alkaloids are particularly interesting due to their diverse biological roles such as pigments and toxins [41]. For instance, ergot alkaloids are highly toxic and are used to produce lysergic acid diethylamide (LSD), but also against different degenerative diseases [55]. Therefore, fungal alkaloids have been mostly studied in search of further drugs that can be used in the pharmaceutic industry.

#### 2.2.2. Ecological Role of Specialized Metabolites

##### Deterrents and Self-Protective Compounds

Fungi have evolved a variety of defense strategies in order to protect themselves against adverse abiotic (e.g., UV radiation) and biotic environmental conditions (e.g., feeding animals, infections by bacteria, other fungi and viruses) [7,13]. For instance, melanin, which is an indolic polymer, and other pigments, which are typically found in spores and hyphae, protect fungi against UV damage and antioxidant chemicals [13,57]. Fungi have developed compounds with bitter or pungent properties (e.g., some indole alkaloids) that can be also in some occasions linked to toxicity [6,40], and can deter potential predators and pathogens, or overtake the defenses of potential preys and host organisms. Indeed, it has been shown that fungivorous animals can learn to avoid fungi with pungent properties, such as the case of the opossum *Didelphis virginiana*, who learned to avoid fruiting bodies containing the toxin muscimol [58,59,60]. Furthermore, fungal volatile compounds can act as repellents, e.g., 1-octen-3-ol is a deterrent of the banana slug *Arioliomax columbianus* from consuming the mushroom *Clitopilus prunulus* [61]. Many fungal metabolites act as toxins, therefore they are known as mycotoxins, and further classify as aflatoxins, ochratoxins, zearalenone, trichothecenes, cytochalasins and fumonisins [40]. Although their ecological role in nature has been rarely tested, it is reasonable to think that they evolved as a mean to protect fungi against potential predators and overcome the defenses of potential preys.

##### Chemical Communication in Ecological Associations, and Mutualistic and Symbiotic Interactions

Fungi evolved diverse associations with fungal and non-fungal organisms. Fungi communicate and interact either via VOCs or secreted substances [14,15]. Examples of metabolic exchange include cell surface recognition, quorum-sensing, biofilm formation and secretion of antibiotics, among others [62,63,64]. Each of these types of interaction plays a vital part in the fungi metabolic exchange and provides the basis for their survival. VOCs also play a major role in intraspecific interactions as they trigger certain behaviors such as attraction between gametes for mating in sexual reproduction, sporulation and induction of morphological changes. For instance, in ascomycetes and basidiomycetes, there are metabolites that have a function of mating pheromones, which can allow the discrimination among fungi genotypes [65,66]. Some fungi attract animals to facilitate the dispersion of their spores; this attraction is usually achieved through individual VOCs such as dimethyl sulfide that is released by, e.g., truffles (i.e., *Tuber* spp.) to attract mammals and insects [67,68], or mimicking complete blends of VOCs such as the parasitic *Puccinia monoica*, that mimics the scent of co-occurring plants [69]. Both VOCs and non-VOCs also participate in the communication within strong mutualistic associations such as plants and mycorrhizal fungi. Metabolites of mycorrhizal fungi are able to trigger plant defense responses [40,70]. Fungi also seem to have a predominant role in the ecological interactions of lichens, symbiotic associations of fungi and algae organisms, as most of the lichen specialized metabolites are from fungal origin. These lichen metabolites (e.g., atranorin, usnic acid and vulpinic acid) may act as allelopathic compounds, repellents of potential herbivores and protective against radiation, among others [71,72].

## 3. Common Mass Spectrometry-Based Strategies Used in Mycology Studies

Mass spectrometry tools enable the analysis of multiple molecules from a complex sample; those identified chemical compounds can be then correlated to a relevant phenomenon or biological question. Thus, applications of MS in mycological studies are not restricted to the identification of chemical compounds, but it is also used to perform fungi identification and classification, especially of micro-fungi. In the following two sections, we show the most common uses of MS-based studies in mycology (see also Table 1).

### 3.1. Biotyping: Microbial Classification and Identification

Rapid and reliable biological identification, especially for pathogens, is crucial in clinical diagnosis for prescribing the most efficient treatment. In fact, biotyping is a type of chemotaxonomy. Chemotaxonomy is a biological classification based upon the analytical measurement of chemical constituents, either as unique biomarkers or as contributors to a fingerprint profile (e.g., distinctive ratios of chemical components). Hence, biotyping is a potential alternative to genetic-based taxonomy, when there are no genome information nor genetic sequencing facilities available [64,151,152]. Mass spectrometry has been routinely used in chemotaxonomic studies by utilizing MS data from known microbes as reference data (e.g., using internal standards or reliable databases) [78,153,154]. In particular, matrix-assisted laser desorption/ionization time-of-flight mass spectrometry (MALDI-TOF MS) has emerged as a tool for the accurate, cost-effective, sensitive and robust biotyping of microorganisms in daily routine clinical microbiology [73,74,75,76,77,78,79,80,85,86,87,88,89,90,91,92].

The analysis of protein profiles via MALDI-TOF MS can help to differentiate and/or identify unknown microorganisms at the genus and even species level by matching microbial mass spectra against spectral libraries collected from known organisms. Taxonomical identification based on protein profiles has been reliably achieved at the species level in some fungi, such as *Aspergillus*, *Candida*, *Cryptococcus*, *Galactomyces*, *Microsporum*, *Penicillium*, *Rhodotorula*, *Saccharomyces* and *Trichosporon*, among others [73,74,75,76,77,78,79,80,81,82,83,85,86,87,88,89,90,91,92,155]. Biotyping has also been found useful in describing new fungal species [156]. However, whether MALDI-TOF MS data are appropriate for identification and discrimination below the species level (e.g., strains) is still controversial [157]. Nevertheless, new approaches and rapidly increasing data bases are in constant development to further increase the resolution of biotyping. The Biotyper (Bruker, Bremen, Germany) and SARAMIS (AnagnosTec, Potsdam-Golm, Germany) databases, which are associated to their respective Bruker Daltonics (Bruker, Bremen, Germany) and Shimadzu (Shimadzu-Biotech Corp., Kyoto, Japan) MALDI-TOF MS systems, have been widely applied in fungal identification [75,76,84,88,157,158]. Samples of undigested cell extracts are most commonly used for MALDI-TOF MS biotyping [152,153,156,159]. Independently of the sample preparation used, the MS measurement is performed in the range of 2 and 20 kDa for the identification of unique ribosomal protein profiles, which are later matched to the reference database. A high advantage of protein fingerprints is that they are constantly expressed; furthermore, protein biotyping is highly reproducible and mostly independent of the culture medium, incubation temperature and growth state [153,159].

Another strategy for MS biotyping is based on VOCs emitted by the fungi. Exploratory analysis of VOCs emitted by fungi typically involves a headspace analysis of in vitro cultures. VOCs-based biotyping has been used to identify species of *Candida* [93,94,98], *Penicillium* [123], *Fusarium* [124] and *Rhizoctonia* [125]. This type of biotyping can employ headspace absorptive extraction techniques associated with gas chromatography-time flight mass spectrometry (GC-TOF-MS) or liquid–liquid extraction methods associated with gas chromatography-mass spectrometry electron ionization (GC-MS-EI). Unlike biotyping using protein profiles, the comparison of VOCs profiles should be carried out under the same conditions to be reliable: this means that they should be sampled in similar conditions (e.g., similar growth conditions) and using the same sampling method because specialized metabolite production is sensitive to diverse factors such as interaction with other microorganisms, environmental contaminants or stress and sampling techniques [14,128,160,161].

### 3.2. Identification of Microbial Chemical Compounds

**Figure 2:**
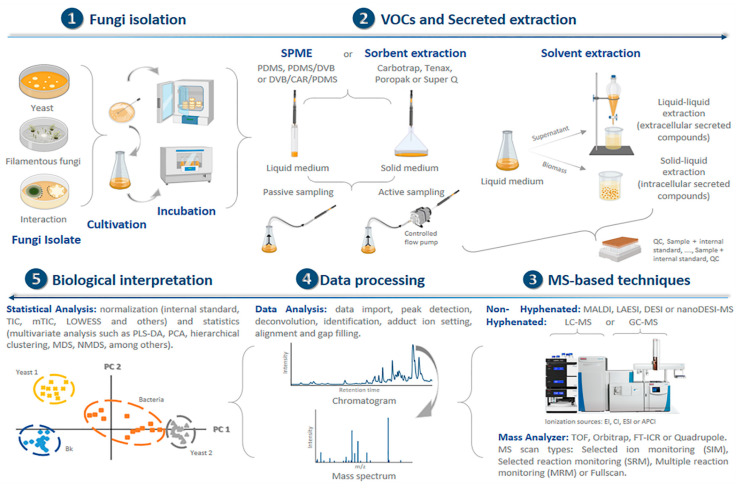
Workflow of studies involving mass spectrometry and identification of fungal chemical compounds. A summary of the steps involving mass spectrometry used in microfungal chemical studies, from sample preparation to analysis. Micro-fungi are used as an example and sample techniques are also shown.

Sampling strategies in metabolomics and the minimization of unwanted sources of variation are highly important. The source of variation can be broadly summarized as: (i) sample collection (including environmental factors) and culture conditions, (ii) metabolite extraction or sample pre-treatment prior to analysis, (iii) parameters of the MS-based instrument and (iv) data processing and compound identification [162,163,164]. The omission or minimization of such unwanted variation can have negative impacts on the development of the study, resulting in, for example, ambiguous identification or the identification of fewer biomarkers if we use non-hyphenated MS, i.e., MS that is not coupled to an orthogonal separation system [162,165,166]. In Figure 2, we summarize the steps involved in metabolomic studies from the preparation of the fungi material through to sampling and MS analysis to data processing and interpretation.

#### 3.2.1. Identification of VOCs of Fungal Origin

At the moment, there are many publications related to fungal volatilomics, and to the best of our knowledge, there are two public VOC databases that include volatile information of more than 10,000 fungal species [45,160,167]. In order to monitor VOCs produced by micro-fungi, it is relevant to remember that VOCs are partitioned between gas and liquid phases under optimal growth conditions. Due to the large diversity of these compounds, there is no “one” analytical protocol to study them all. Gas chromatography coupled to mass spectrometry (GC-MS) is an extremely reliable and affordable method for the identification and quantification of VOCs. In general, VOCs are collected from a headspace, trapped on an adsorptive surface or solvent extracts, followed by thermal desorption or direct injection into the GC for separation, and then the individual VOCs from complex mixtures are identified by comparisons of the mass spectra with databases, internal standards and/or chromatographic retention indices [14,99,115,128,129,168,169]. Other mass spectrometry methods that have been used for the identification of VOCs include real-time measurements, which omit the use of a chromatography technique, such as proton transfer reaction-mass spectrometry (PTR-MS) that, for example, has been used in measuring volatile emissions of *Ascocoryne sacroides* (100), *Hypoxylon* sp. [99], *Muscodor albus* [101] and *Nodulisporium* sp. [102]. Another direct MS technique is selected ion flow tube-mass spectrometry (SIFT-MS) that has been adapted for the detection of volatile traces from medically important fungi such as species of *Aspergillus*, *Candida*, *Cryptococcus*, *Fusarium* and *Mucor* [103,104,105]. Secondary electrospray ionization coupled to high-resolution mass spectrometry (SESI-MS) has also been used to analyze the VOCs produced during fermentation by the ale yeast *Saccharomyces cerevisiae* [106].

An exquisite advantage of the PTR-MS, SIFT-MS and SESI-MS online approaches is their relative simplicity [104,170,171], where small molecules can be directly detected from the volatile emissions of the samples of interest. Hence, the VOCs can be easily monitored in real-time and can continuously collect data about specific compounds [100]. However, online approaches have two major disadvantages when compared with offline methods (e.g., GC-MS; HPLC-MS): (i) a lack of resolution of isobaric compounds (i.e., compounds with identical mass-to-charge ratios), and ii) sensitivity to matrix effects [106,172]. In general, MSs that are not coupled to an orthogonal separation system may provide ambiguous identification of VOCs [162,165].

Though gas chromatography remains an excellent method for the separation of isobaric VOCs, this method is time-consuming. An alternative separation method of isobaric VOCs is ion mobility coupled to mass spectrometry (IMS). Ion mobility separation has a different principle [173,174] than the one used in GC or LC-MS; hence it is able to resolve isobaric species and provide the rapid, sensitive and broad spectrum detection of trace organic components in moderately complex gas mixtures such as those found in *Aspergillus* species [130].

The collection of volatile specialized metabolites can be performed by direct measurement, as in the case of PTR-MS, SIFT-MS and SESI-MS, or by using a direct collection system such as headspace techniques. Unfortunately, these collection strategies can be influenced by the volatiles that are also emitted by the cultivation media or by other sources in the environment during the analysis. These additional volatiles can distort the signal profiles that are detected on the mass spectrometer [103,162,164]. Therefore, the use of a suitable sample extraction strategy is recommended such as solid phase micro extraction (SPME), which involves a metallic fiber that has been coated with different adsorbent materials. Coating materials include divinylbenzene (DVB), polydimethylsiloxane (PDMS), carboxen (CAR), polyacrylate (PA) and carbon wide range (CWR). These coatings have been used solely or in combination (Table 1) [113,130,163]. Hence, SPME-based sampling consists of the preconcentration of the VOCs on a fiber via absorption, and the later thermal desorption (delivered) of the captured volatiles to the GC column or directly to the mass spectrometer instrument (see Figure 2) [114,128]. 

Volatiles can also be collected by solvent extraction or liquid–liquid extraction, when they have not yet been released to the environment and therefore not yet in the gas condition. The selection of the solvent depends mainly on the chemical class of VOCs [165,169]. For example, pentane and hexane can be used, although ethyl acetate and dichloromethane are also commonly used, as solvents for the extraction of volatile compounds of fungal species. Independently of the method used for volatile collection, it is important to consider that crucial differences in the VOC profile will emerge from biological factors, such as the different emission of VOCs due to fungi cultivation on solid or in liquid medium [15,160,163,175]. These differences in VOC profiles arise most likely due to the different cell numbers and varying diffusion of oxygen into the medium [176]. Other factors that can play a role in VOC profiling are the cultivation temperature, time points at which samples are collected and humidity, among others [177]. Furthermore, VOC profiles will depend on the air sampling collection technique used [99,115,116,117,129,165,169,178]. Air sampling collection can be classified into two categories: active and passive. Active air sampling (AAS) techniques have been considered the most accurate way to measure VOC concentrations in air. These methods exploit a pump or vacuum to force analytes present in the air to flow through a sampler. The sampler can be an SPME or another trapping surface. Passive air sampling (PAS) techniques do not use a driving force, they work based on permeation or diffusion [14,175].

#### 3.2.2. Identification of Non-Volatile Compounds of Fungal Origin

Due to the chemical diversity of non-volatile compounds that are secreted/produced by fungi, it is practically impossible to extract quantitatively all specialized metabolites using a single sample preparation method. Consequently, sample preparation is an important aspect of specialized metabolite profiling and will, independently of the method, lead to a bias toward certain types of compounds. For example, for the extraction of non-polar components, such as lipids, polyketides and terpenoids, one may use water-immiscible solvents such as ethyl acetate or dichloromethane. Other water-immiscible solvents such as ethers, chloroform and carbon tetrachloride are less common due to their toxicity and negative environmental effects [134,179]. Additionally, pH is crucial for the extraction, due to ionizable moieties in the chemical structure of the metabolites being extracted into the organic phase to a much higher degree in their neutral form than in a charged state. In most of the cases is a low pH extraction necessary, but it can be replaced in some cases with a neutral extraction (e.g., for stability reasons) [118,165].

To extract polar components, one may use acetone, methanol and ethanol [143,144,150]. The most common solvent used to extract polar intracellular secreted compounds is methanol (see Figure 2). Nevertheless, it has been reported that methanol and ethanol extraction can yield in the extraction of non-polar waxes, sterols, and triglycerides, as well. Thus, it is necessary to perform a second extraction step, such as extraction with ethyl acetate [118,165].

A major obstacle in any sample preparation is the possibility of unwanted chemical reactions taking place, such as precipitation, complex formation and degradation, among others. To reduce this risk, fast extraction procedures are preferred. This means extraction procedures of 10 or fewer minutes [118,165,166]. One of the most common methods used to achieve the best separation of secreted specialized metabolites produced by fungi is reverse phase (C-18) chromatography since it is more than ideal to interact with polar and some non-polar metabolites [150,180,181,182]. In some cases, before injecting samples on LC, samples are needed to be purified on small solid phase extraction (SPE) columns to remove chromatography-impairing lipids and phospholipids [147,148,183].

Interestingly, performing liquid separations at low pH (by addition of formic or acetic acid) is statistically better since sharper peaks can be obtained on the chromatogram. Formic acid and acetic acid are acid additives that are compatible with mass spectrometry detection, meanwhile, at the same time, they keep acidic moieties in specialized metabolites protonated. Nevertheless, some metabolites can be affected by the addition of acidifiers or if the mass spectrometry measurement is done in the negative mode [149,184,185].

Other chromatographic techniques use two consecutive separation columns (2-dimensional liquid chromatography) or columns of different sorbent chemistry, such as ionic exchangers (see Table 1) [14,113,129,134,143]. The most common ionization techniques used in combination with 2D liquid chromatography are the ambient pressure ionization techniques, such as electrospray ionization (ESI) [134,135,137,138,140,141,142,143,144,145,150] and atmospheric pressure chemical ionization (APCI) [139,146]. These are all soft ionization techniques (i.e., they produce limited chemical fragmentation). Hence, mass spectrometry signals are extremely clean and easily interpretable.

The possibility of adducts formations as well as multiple charge metabolites can make researchers disagree with the “easily interpretable” statement [131,140,146]. However, new high-resolution mass spectrometers, which can perform tandem MS fragmentation and analysis, can improve the correct identification of the specialized metabolites by decreasing the incorrect chemical composition assignment of the ion signals. Furthermore, specialized data handling software can further decrease the risk of misassignments, since it is capable of analyzing tandem MS fragmentation signals to determine the “chemical” relation among the measured signals, or to search an unknown MS signal in a database by comparing the fragmentation patterns in the tandem MS spectra and the structural information of fungal specialized metabolites [45,139,141,186,187,188]. Today the high-resolution mass spectrometry market is mainly divided by time of flight (TOF), Orbitrap and Fourier transform ion cyclotron (FT-ICR).

Non-volatile compounds produced by fungi can be also monitored and measured in situ. This is particularly helpful, if it is not possible to gain enough fungi biomass or if it is interesting to analyze the spatial distribution of the metabolites in a culture. In this case, direct ionization sources compatible with mass spectrometry imaging (MSI), such as MALDI [107], laser electrospray ionization (LAESI) [131,189,190,191], desorption electrospray ionization (DESI) [108,109,110,111] and nano desorption electrospray ionization (nanoDESI) [110,131,192], can be directly used for examining fungal cultures such as for species of *Clohesyomyces*, *Fusarium*, *Moniliophthora*, *Penicillium* and *Trichoderma*.

Nevertheless, all the above-mentioned methods have limitations [110,131,193]. For example, typical surfaces observed in fungal cultures can be detrimental to DESI, since viscous biofilms can clog the spraying systems. Furthermore, MALDI- or LAESI-based MSI systems may have trouble achieving optimum ablation/ionization signals due to the lack of a constant laser focal distance to the sample surface, since fungal surfaces often possess complex topography because of the presence of aerial hyphae [109]. All MSI techniques may also suffer from misidentification in the case of isobaric species [161,173]. For several years, ion mobility instruments have been used to improve metabolite identification, in particular for MSI experiments. However, up today, we have not yet found a hybrid IMS-MS imaging experiment performed on fungi. We still believe that in the following years, better MSI instrumental setups would overcome the above-mentioned challenges.

## 4. Future Directions

The applications of MS in mycology are very promising. The recent developments in mass spectrometry imaging have dramatically facilitated and improved the identification of fungi species. However, to elucidate the ecological role of fungi in communities, as well as understand their ecological interactions with other group of organisms such as algae or bacteria, it is necessary to identify the metabolites they produce. Mass spectrometry has also improved the identification of fungal specialized metabolites; however, the identification reliability still needs to improve. The main challenge is still the limited number of dedicated fungi libraries and repositories containing MS data of specialized metabolites, where all chemical and taxonomic information have been merged together. As reported here, newer sample handling protocols as well as ionization sources that have been successfully applied to human, plants and animal studies have yet to migrate to mycology studies. One challenge is the wide fungi diversity as well as the lack of universal sample handling/MS protocols. Therefore, collaborative efforts may be needed to increase or enrich the MS libraries and consequently improve the reliability of the identification of specialized metabolites, as well as specific identification. Better repositories of specialized metabolites produced by fungi will then shed light on the molecular adaptations of the fungi to their environment and on their interactions with other organisms of the community. The idea is to treat the fungi and the community as a whole, integrating the fundamental biological knowledge, to obtain an understanding of every particular ecosystem for the purpose of biotechnological applications.

## Figures and Tables

**Table 1:** Mass spectrometry-based strategies used in mycological studies.

Application	MS System	Associated MS Database	Sample Processing	Targeted Chemical Compounds	References
Biotyping	MALDI-TOF-MS, MALDI-TOF-IC MS	Biotyper, SARAMIS, MBT Compass, BDAL	solvent extraction (ethanol-formic acid, trifluoroacetic acid or formic acid/acetonitrile)	peptides or proteins	[73,74,75,76,77,78,79,80,81,82,83,84,85,86,87,88,89,90,91,92]
Biotyping	Rapid evaporative ionization mass spectrometry (REIMS)	In-house database	no pre-processing	fatty acids, lipids	[93,94]
Biotyping	PCR electrospray ionization-mass spectrometry (PCR/ESI-MS)	PLEX-ID system, GenBank	no pre-processing	proteins, polysaccharides	[95,96,97]
Biotyping	Paper spray mass spectrometry (PS-MS)	In-house database	disruption analysis (cell lysis method)	lipids	[98]
Metabolomics	PTR-MS	NIST library, chemical standards	no pre-processing	alcohols, aldehydes, esters, ketones, lipids, organic acids, terpenes, among other VOCs	[99,100,101,102]
Metabolomics	SIFT-MS	Precursor–VOC reaction	no pre-processing	alcohols, aldehydes, ketones, among other VOCs	[103,104,105]
Metabolomics	SESI-MS	In-house database	no pre-processing	fatty acids, among other VOCs	[106]
Metabolomics	LDI-MS	In-house database	solvent extraction (e.g., dichloromethane)	anthraquinones, aromatics, chromones, depsides, depsidones, dibenzofurans, diphenylethers, lipids, naphthoquinone, polyols, xanthones.	[107]
Metabolomics	DESI-MS	In-house database	no pre-processing	anthraquinones, peptaibols, phomopsinones, pyrroloindoles	[108,109,110,111]
Biotyping	Gas chromatography-combustion-isotope ratio mass spectrometry (GC-C-IRMS)	In-house database, a mixed raster of 14 amino acids with known ẟ-13C	solvent extraction (e.g., dichloromethane)	amino acids	[112]
Biotyping and Metabolomics	GC-MS, GC-IMS, GC-FID Ionization sources: EI, CI, APCI Mass analyzer: Q or TOF	NIST library, LTPRIs, chemical standards	-liquid–liquid extraction (ethyl acetate–methanol, ethyl acetate–water) -solid–liquid extraction (methanol, dichloromethane or methanol-chloroform-water) -passive sampling, SPME (PA, PDMS, DVB, CAR/PDMS, DVB/PDMS or DVB/CAR/PDMS), SPE (C18) or sorbent (PTFE, Carbotrap, Tenax or bentonite) -active sampling, sorbent or SPME	alcohols, aldehydes, alkanes, alkenes, amino acids, carboxylic acids, carbohydrates, esters, ketones, fatty acids, lipids, organic acids, terpenes, among other VOCs	[14,99,100,102,113,114,115,116,117,118,119,120,121,122,123,124,125,126,127,128,129,130]
Metabolomics	LC-MS	ACD IntelliXtract, in-house database	direct analysis (droplet−LMJ−SSP)	anthraquinones, alkaloids	[131,132]
Metabolomics	LC-MS Ionization sources: ESI or APCI Mass analyzer: ion-trap, orbitrap, Q, TOF	Antibase, LDB, GNPS, ACD Chemfolder, NIST, DNP, Mycosynthetix, ORFs, Peptaibol, chemical standards	-liquid–liquid extraction (ethyl acetate, methanol-chloroform or ethyl acetate:acetic acid) -solid–liquid extraction (acetone, methanol, dichloromethane or dichloromethane:methanol) -cleanup by SPE (C18)	anthraquinones, aromatics, chromanones, chromones, depsides, depsidones, dibenzofurans, diphenylethers, lipids, naphthoquinones, peptides, polyols, xanthones.	[133,134,135,136,137,138,139,140,141,142,143,144,145,146,147,148,149,150]
